# Qualitative and Quantitative Evaluation of the Image Quality of MDCT Multiphasic Liver Scans in HCC Patients

**DOI:** 10.1155/ijbi/4163865

**Published:** 2025-01-16

**Authors:** Mohamed Zakaria El-Sayed, Mohammad Rawashdeh, Hend Galal Eldeen Mohamed Ali Hassan, Mohamed M. El Safwany, Islam I. E., Yasser I. Khedr, Moustafa A. Soula, Magdi A. Ali

**Affiliations:** ^1^Medical Imaging Sciences Department, College of Health Sciences, Gulf Medical University, Ajman, UAE; ^2^Faculty of Applied Medical Sciences, Jordan University of Science and Technology, Irbid, Jordan; ^3^Diagnostic and Interventional Radiology and Molecular Imaging Department, Faculty of Medicine, Ain Shams University, Cairo, Egypt; ^4^Technology of Radiology and Medical Imaging Program, Faculty of Applied Health Sciences Technology, Galala University, Suez 435611, Egypt; ^5^Radiology and Medical Imaging Department, Faculty of Applied Health Sciences Technology, Pharos University in Alexandria, Alexandria, Egypt; ^6^Physics Department, Faculty of Science, Damanhour University, Damanhour, Egypt; ^7^Medical Imaging and Radiography Department, Faculty of Allied Medical Sciences, Aqaba University of Technology, Aqaba, Jordan

**Keywords:** CT, image quality, qualitative assessment, quantitative assessment

## Abstract

**Background:** The quality of CT images obtained from hepatocellular carcinoma (HCC) patients is complex, affecting diagnostic accuracy, precision, and radiation dose assessment due to increased exposure risks.

**Objectives:** The study evaluated image quality qualitatively and quantitatively by comparing quality levels with an effective radiation dose to ensure acceptable quality accuracy.

**Materials and Methods:** This study retrospectively reviewed 100 known HCC patients (Li-RADS-5) who underwent multidetector computed tomography (MDCT) multiphasic scans for follow-up of their health condition between January and October 2023. The evaluation involved quantitative and qualitative analyses of parameters such as SD, SNR, and CNR, as well as a qualitative assessment by two radiology consultants. The outcomes were compared, and the effective dose was calculated and compared with both quantitative and qualitative assessments of image quality.

**Results:** ROC curve analysis revealed significant differences in CT image quality, with high to moderate specificity and sensitivity across all the quantitative parameters. However, multivariate examination revealed decreasing importance levels, except for CNR (*B*, 0.203; *p* = 0.001) and SD BG (*B*, 0.330; *p* = 0.002), which increased in *B*. The CNR and SD BG remained independent variables for CT image quality prediction, but no statistically significant relationship was found between the effective dose and image quality, either quantitatively or qualitatively.

**Conclusion:** This study underscores the vital role of both quantitative and qualitative assessments of CT images in evaluating their quality for patients with HCC and highlights the predictive importance of CNR, SNR, and SD. These findings emphasize the value of these devices in assessing and predicting outcomes to minimize the effective dose.

## 1. Introduction

Hepatic multiphase CT is widely used to evaluate focal liver lesions in patients with chronic liver disease or liver cirrhosis [1]. This imaging technique is typically employed for detecting HCC or monitoring posttreatment follow-ups involving four phases: precontrast, hepatic–arterial, portal–venous, and delayed. Patients with liver cirrhosis or chronic liver disease often need to undergo multiple CT scans throughout their follow-up care. Therefore, understanding the minimum radiation dose essential for detecting HCC via hepatic multiphase CT scans holds the potential to reduce radiation exposure in patients included in these studies [2].

The expanded use of CT across diverse settings and varying vendor platforms has raised concerns regarding radiation exposure, potentially leading to increased risks of malignancy. Consequently, concerted efforts are underway to minimize radiation doses associated with CT scans while concurrently striving to uphold or even enhance image quality. These endeavors heavily rely on advancements in CT scanner technologies and software innovations [3–5].

Within the realm of oncology imaging, strategies aimed at reducing the dose may adopt a more constructive approach, prioritizing the improvement of image quality at standard radiation doses, despite the potential advantages [6]. Hence, there is strong advocacy for the standardization and optimization of clinical protocols. This ensures that the radiation dose remains as low as reasonably achievable (ALARA) while simultaneously preserving diagnostic certainty and maintaining high image quality [7].

CT image quality assessment often relies on subjective interpretation by two radiology consultants, introducing inherent variability influenced by individual preference and experience. This subjectivity, coupled with interreader variability, poses challenges for consistent evaluations, both within and between studies. Comparing routinely acquired CT scans with those involving radiation dose reduction systems is intricate due to the subtle differences discernible mainly to the trained eye; to address this, an objective and quantitative assessment of CT image quality parameters such as image, noise, signal-to-noise ratio (SNR), and contrast-to-noise ratio (CNR) facilitates a more standardized approach. Such an approach facilitates comparisons between studies and aids in establishing optimal thresholds for obtaining diagnostically adequate CT images. However, these evaluations often focus on specific areas of interest [8].

In our retrospective study, our objective was to evaluate the image quality of patients diagnosed with HCC. The intent was to establish accepted standards of image quality and subsequently compare these benchmarks with the effective radiation dose.

## 2. Materials and Methods

### 2.1. Ethical Considerations

Our retrospective study was approved by the medical ethics committee of Pharos University in Alexandria, Egypt.

We conducted a retrospective review involving 100 adult patients with available clinical history, radiological reports, and confirmed diagnoses who were followed up on their known diagnosis of HCC (Li-RADS-5) between January and October 2023. The study involved MDCT multiphasic liver imaging performed using a multislice CT scanner. CT scans were traced from the picture archiving and communication system (PACS) Advantage Workstation for Diagnostic Imaging ADW 4.7 using the patient's medical record number (MRN). The data collected consisted of patient age, sex, weight, date of MDCT image acquisition, contrast protocol, and imaging protocol. The study included the patient's previous radiological examinations, such as ultrasonography, fibroscan, and laboratory investigations. Our inclusion criterion included participants diagnosed with HCC (Li-RADS-5). Conversely, individuals diagnosed with liver metastases or abscesses were excluded from the study.

### 2.2. CT Image Acquisition

Patient data were obtained via multiphasic CT of the liver using the same MDCT system. Specifically, General Electric HealthCare Optima CT scanners were used. A dedicated multiphase protocol involving a collimation of 1.5 mm and an image reconstruction interval of 3 mm was used. Dynamic studies include a sequence of unenhanced images followed by enhanced images captured during the hepatic arterial, portal, and delayed phases [9]. To optimize the scan time, computer-assisted bolus-tracking software was used. The acquisition of arterial phase images commenced at 12 s after the automatic detection of aortic peak enhancement, set at 120 Hounsfield units (HU). Subsequently, portal-phase images were acquired 55 s after the peak enhancement.

### 2.3. Quantitative Assessment

All the quantitative assessments were conducted using a CT machine (General Electric HealthCare Optima CT scanners and their dedicated workstation). The image sets were presented using a predefined soft tissue window with a window width (WW) of 400 HU and a window level (WL) of 40 HU.

In the assessment of image quality, our methodology incorporated five specific metrics:

1. Standard deviation (SD) of the background (BG) in the subcutaneous fat of the abdominal wall
2. The SD which was measured within the liver lesion
3. SNR calculation pertaining to the liver lesion images
4. The SNR which is derived from the BG in the subcutaneous fat of the abdominal wall
5. The CNR which specifically assesses the relationship between the liver lesion and the subcutaneous fat of the abdominal wall

These metrics were meticulously selected to provide a comprehensive evaluation encompassing critical parameters contributing to image quality in our study [10].

**Figure 1:**
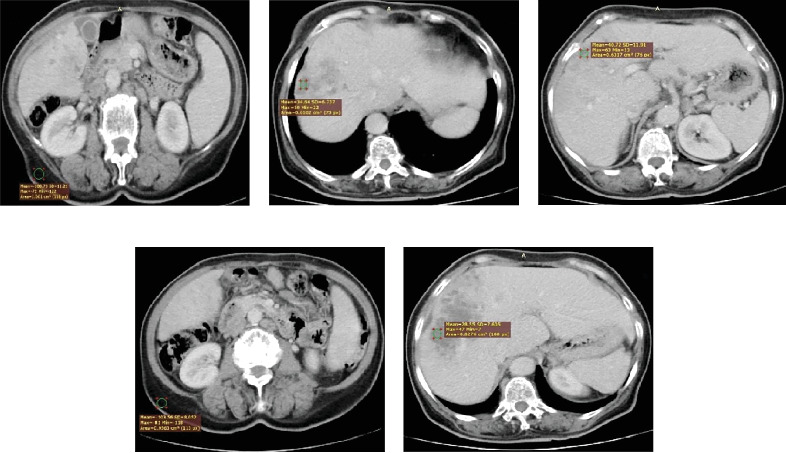
(a) Measurement of the SD BG for subcutaneous fat in the abdominal wall, (b) measurement of the SD for liver lesions, (c) measurement of the mean attenuation of liver lesion ROI_1_ and the SD_1_ for image noise, and (d) measurement of the mean attenuation of subcutaneous fat in the abdominal wall and the SD_*o*_ of image noise. (e) Measurement of the mean attenuation of the liver lesion in ROI_1_.

Objective image noise was quantified by assessing the SD BG of the pixel values within a defined region of interest (ROI_*ο*_, 0.5 : 1 cm^2^) manually drawn by two radiology consultants on a homogenous region of subcutaneous fat of the abdominal wall (Figure 1(a)). The measured SD BG is reported in HU [11].

The liver lesion SD of attenuation coefficient values was measured for images as the liver lesion SD of the pixel values from ROI_1_ (0.5 : 1 cm^2^) manually drawn by two radiology consultants on a region of the liver lesion (Figure 1(b)). The liver lesion SD was measured in HU.

SNR values for the liver lesion images were calculated by dividing the mean attenuation of the liver lesion ROI_1_ by the SD_1_ of image noise for ROI_1_ in the liver lesion, as shown in Figure 1(c). The SNR was calculated by Equation (1) [12]:

(1) $$\begin{array}{c} \text{SNR}=\frac{{\text{ROI}}_{1}}{{\text{SD}}_{1}} \end{array}$$

where ROI_1_ is the mean attenuation of the liver lesion and SD_1_ is the SD of ROI_1._

The CNR relative to the liver lesion for the subcutaneous fat of the abdominal wall is shown in Figures 1(d) and 1(e) and was calculated using Equation (2):

(2) $$\begin{array}{c} \text{CNR}=\frac{{\text{ROI}}_{1}-{\text{ROI}}_{ο}}{{\text{SD}}_{ο}} \end{array}$$

where ROI_1_ is the mean attenuation of the liver lesion, ROI_*ο*_ is the mean attenuation of BG in the subcutaneous fat of the abdominal wall, and SD_*ο*_ is the image noise for ROI_*ο*_ in the subcutaneous fat of the abdominal wall [13].

### 2.4. Qualitative Assessment

Qualitative image quality was evaluated separately by two readers, a diagnostic radiology consultant at the Faculty of Medicine at Alexandria University and the Faculty of Applied Health Science at Pharos University, Egypt, who have great experience in the diagnosis of multiphasic MDCT abdominal imaging for more than 15 years (averaging 1500 cases per year) on a dedicated Advantage Workstation for Diagnostic Imaging (ADW 4.7), for each patient and in all reconstructed datasets. Both readers are aware and knowledgeable about the clinical information and diagnosis of each patient. Images were evaluated using a 4-level scoring scheme: 1 = unacceptable image noise (poor), 2 = average image noise affecting the image (moderate), 3 = minimal image noise (good), and 4 = high-definition (HD) image (excellent), as demonstrated in Table 1 [14].

The images were reviewed visually by two radiology consultants, who were blinded to patient demographic data and CT parameters. The images were initially displayed on a preset soft-tissue window (WW, 400 HU; WL, 40 HU), and the readers were granted the flexibility to adjust the window settings as needed. Both readers were tasked with assessing image quality using a 4-level scoring scheme previously reported in the literature [15].

### 2.5. Statistical Analysis of the Data

The data were analyzed using the IBM SPSS Software Package Version 20.0. Categorical data are presented as numbers and percentages. For continuous data, the normality of the data was tested by the Kolmogorov–Smirnov test. Quantitative data are expressed as the range (minimum and maximum), mean, or Pearson coefficient to indicate the correlation between two normally distributed quantitative variables. The Kruskal–Wallis test was used for comparisons of abnormally distributed quantitative variables between more than two studied groups, and post hoc Dunn's multiple comparison test was used for pairwise comparisons. The Spearman coefficient was used to assess the correlation between two abnormally distributed quantitative variables. A receiver operating characteristic (ROC) curve [16, 17] was generated by plotting sensitivity (TP) on the *y*-axis versus 1 − specificity (FP) on the *x*-axis at different cutoff values. The area under the ROC curve denotes the diagnostic performance of the test. Areas greater than 50% exhibit acceptable performance, and areas of approximately 100% yield the best performance according to the test. The ROC curve also allows a comparison of performance between two tests. Regression was used to detect the most independent factor affecting the total number of readers. The significance of the obtained results was judged at the 5% level.

## 3. Results

### 3.1. Qualitative Assessment

Table 2 shows the means and SDs of the maximum and minimum values of the image noise level score determined by two expert radiology consultants according to qualitative imaging criteria. The results are presented as the mean ± SD and median (min–max) 6.59 ± 1.39 and 6.5 (3.5–10), respectively. The qualitative assessment was also performed based on the image criteria of precision and accuracy, which contribute to an accurate diagnosis. The percentages of excellent, good, moderate, and poor image quality were 7 (7%), 27 (27%), 61 (61%), and 5 (5%), respectively.

### 3.2. Quantitative Assessment

**Figure 2:**
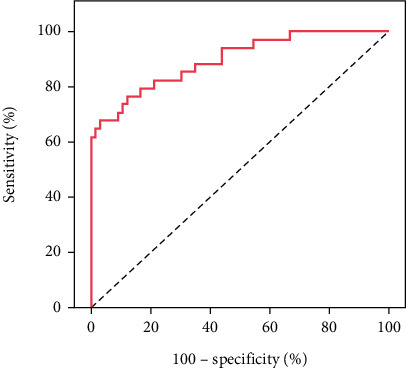
ROC curve for the CNR in predicting good image quality.

**Figure 3:**
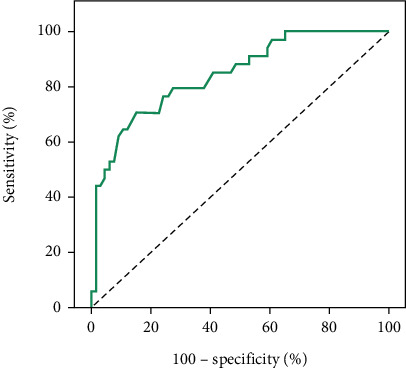
ROC curve for the SD BG for predicting good image quality.

**Figure 4:**
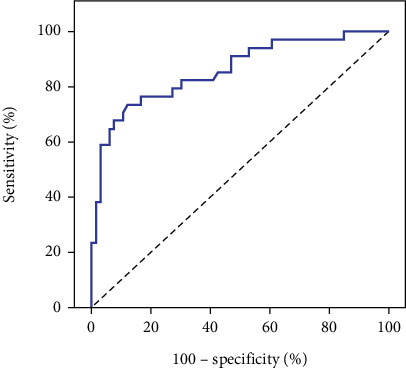
ROC curve for the BG SNR for predicting good image quality.

**Figure 5:**
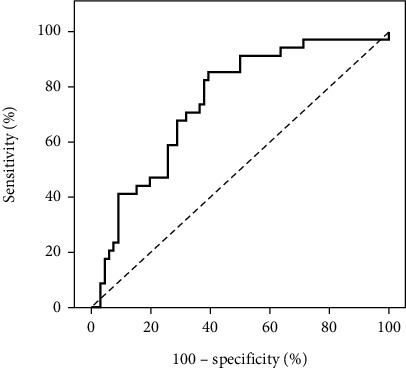
ROC curve for the ability of the SNR to predict good image quality.

The ROC curve analysis was used to assess the sensitivity and specificity of quantitative parameters (CNR, SD BG, BG SNR, and liver SNR) in differentiating quality from nonquality CT images. Statistically significant differences were found for each parameter (*p* values: 0.001, 0.001, 0.001, and 0.002) with cutoff points of > 26, ≤ 7.7, > 11.63, and > 14, respectively. The models demonstrated good to fair specificity and sensitivity for all parameters, as shown in Table 3 and Figures 2, 3, 4, and 5.

Univariate and multivariate analyses were performed on the quantitative parameters to identify factors that correlated with the predictive quality of the CT images using Cox proportional hazard regression model analysis.

Univariate logistic regression analysis identified significant associations between CT image quality and quantitative parameters. The analysis found that CNR, SD BG, BG SNR, and liver SNR were independent variables predicting CT image quality, as shown in Table 4.

According to multivariate analysis of the studied quantitative parameters, the CNR and SD BG were significant regardless of the other adjusted quantitative parameters. Neither the BG SNR nor the liver SNR could independently predict the quality of CT images.

In general, the multivariate examination showed a decrease in importance levels (*B*), except for the CNR and SD BG, which increased in importance. The CNR and SD BG remained to be independent variables for predicting the quality of CT images (CNR (*B*, 0.203; *p* = 0.001) and SD BG (*B*, 0.330; *p* = 0.002)).

The CNR and SD BG were found to be independent variables for constructing predictive models. The CNR and SD BG demonstrated significant differences in quality between CT images, as shown in Table 3.

**Figure 6:**
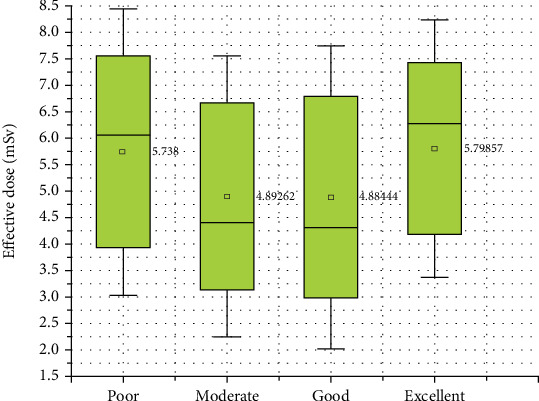
Correlation between the effective dose and image quality.

This study showed that there was no statistically significant relationship between the effective dose and image quality, either quantitatively or qualitatively, as shown in Tables 5 and 6 and Figure 6, indicating that the quality of images depends on the optimal use of all aspects of imaging procedures, not just the radiation dose.

## 4. Discussion

CT plays a crucial role in the management of patients diagnosed with HCC. As an imaging modality, MRI provides exceptional contrast resolution and enables effective detection of metastases and precise disease staging [18].

Reducing CT radiation doses in cancer patients, especially those with HCC, is crucial for several reasons. It helps lower the risk of radiation-induced side effects, such as cataracts and malignant tumors in sensitive organs [19]. Additionally, lower radiation doses allow for safer and more frequent imaging, as high exposure often restricts the number of scans a patient can have [20]. Overall, minimizing radiation doses is key to reducing risks and improving patient care.

The CNR and SNR are pivotal metrics for assessing CT image. CNR specifically measures the discernibility of low-contrast details within CT images by accounting for object contrast and image noise's SD [21, 22]. Moreover, the SNR offers a comprehensive assessment of the entire image [23]. A new physical image quality index is derived from the SNR and provides good approximations for evaluating low-contrast detectability in CT images [24]. These metrics play a critical role in assessing image quality and serve as valuable tools in fine-tuning CT scanning parameters, ultimately enhancing diagnostic precision.

Evaluating CT images both quantitatively and qualitatively offers distinct advantages over solely assessing the effective dose. Quantitative assessment enables the precise measurement of image quality parameters such as contrast and noise levels, offering valuable insights into diagnostic potential [25]. This quantitative approach aids in determining optimal exposure levels for generating diagnostically suitable images [26]. Conversely, qualitative evaluation involves visual interpretation, enriching the understanding of image quality and diagnostic performance [22]. Radiologists, through visual assessment, can detect artifacts or abnormalities, potentially impacting diagnostic accuracy [27]. Overall, both quantitative and qualitative evaluation methods complement the assessment of effective dose, providing specific and detailed insights into CT image quality and diagnostic potential.

In our retrospective study, our primary aim was to assess the diagnostic efficacy of hepatic multiphase CT images in HCC patients, aiming to improve diagnostic accuracy by scrutinizing accepted levels of image quality against corresponding effective doses.

The study's outcomes highlighted the significance of image quality, underscoring its pivotal role in facilitating precise and clear diagnostic interpretations. Specifically, the distribution of image quality ratings was as follows: excellent (7%), good (27%), moderate (61%), and poor (5%). These findings underscore the diversity in image quality representations observed within our study cohort.

The ROC curve serves as a valuable tool for predicting CNR values and establishing cutoff points for assessing CT image quality. Several studies have investigated the relationship between the CNR and observer performance through ROC analysis. One such study revealed a strong correlation between the CNR and observer performance, specifically within a fixed reconstruction filter setting. These findings suggest the applicability of the CNR in predicting low-contrast performance within a limited range of filters [18]. ROC curve analysis plays a crucial role in determining the optimal cutoff value for the CNR in evaluating CT image quality [28]. Additionally, estimating the SNR through the analysis of signal power and noise power from observed images has emerged as a method for evaluating overall image quality [29]. While the ROC methodology has proven beneficial in determining optimal CNR cutoff values for CT image quality assessment, it is essential to acknowledge and consider its limitations in interpretation and application.

The present study's outcomes included the establishment of a ROC curve to discern the sensitivity and specificity of quantitative parameters (CNR, SD BG, BG SNR, and liver SNR) in distinguishing CT image quality from nonquality. Remarkable distinctions were observed in the CNR, SD BG, BG SNR, and liver SNR, identifying respective cutoff points of > 26, ≤ 7.7, > 11.63, and > 14.

Numerous studies have confirmed the association between CT image quality and the CNR and SNR in the liver. Bette et al. highlighted that virtual monoenergetic imaging reconstructions at lower kiloelectronvolt levels notably enhanced the conspicuity of liver metastases, thereby resulting in elevated CNR values [30].

The CNR and SD can be used to predict image quality in CT images of the liver [31]. In a study comparing low-dose liver CT using deep learning denoising (DLD) to standard-dose CT using model-based iterative reconstruction (MBIR), the CNR was significantly greater in the low-dose CT group [32].

In another study, the results indicated that the CNR can be used as a quantitative measure of image quality in CT of the liver and that, at higher CNR values, the sensitivity of detecting liver lesions increases [33].

Bhosale et al. compared the effect of different reconstruction techniques on image quality and reported that images reconstructed with a soft kernel or adaptive statistical iterative reconstruction had higher CNRs than those reconstructed with a standard kernel [34].

The findings of this study underscore the significant associations between CT image quality and several quantitative parameters (CNR, SD BG, BG SNR, and SNR). Univariate logistic regression analysis of the liver revealed the liver. Notably, the CNR (> 26), SD BG (≤ 7.7), BG SNR (> 11.63), and liver SNR (>14) independently predicted CT image quality according to univariate analysis.

Furthermore, the multivariate examination highlighted the CNR and SD BG as independent variables in constructing predictive models, signifying their substantial role in distinguishing between image quality levels in CT scans.

Interestingly, this study revealed no statistically significant relationship between the effective dose and image quality, both quantitatively and qualitatively. To enhance future research and improve imaging practices, it is recommended to increase the sample size to strengthen result reliability and generalizability. Additionally, stratifying patients based on characteristics such as size and weight could reveal important subgroup differences in image quality that were not apparent in the current study. These steps will provide a more nuanced understanding of how various factors impact image quality, leading to more effective optimization of imaging procedures beyond just adjusting the radiation dose.

The present study has several limitations that warrant attention. Firstly, its focus on multiphasic MDCT examinations of the abdominal region restricts its generalizability to a wider population. Secondly, there is a critical need to expand the sample size to include a more diverse patient cohort that is stratified by size and weight. Thirdly, significant challenges arose in effectively integrating a control group into the investigation. Finally, the trend observed in the ROC curve raises concerns about the potential robustness of the method in assessing overall image quality. Therefore, future research should be aimed at addressing these limitations.

In conclusion, our study underscores the importance of integrating quantitative and qualitative evaluations of CT images in assessing HCC patients. The CNR and SD BG emerged as pivotal independent variables for predictive modeling, offering insights into predicting image quality. Implementing these findings could empower clinicians and radiologists to enhance diagnostic potential in multiphasic MDCT examinations.

## Figures and Tables

**Table 1:** Imaging quality scoring criteria.

**Image noise levels**	**Criteria**	**Scores**
Excellent HD	HD image	4
Good	Minimal image noise	3
Moderate	Average noise affecting the image	2
Poor	Above-average noise severely affects the image (unacceptable image noise)	1

**Table 2:** Distribution of the studied patients according to all radiologists and image quality (*n* = 100).

	**No. (%)**
Total readers' scores	
Mean ± SD	6.59 ± 1.39
Median (min–max)	6.5 (3.5–10)
Image quality	
Bad image quality	66 (66%)
Poor	5 (5%)
Moderate	61 (61%)
Good image quality	34 (34%)
Good	27 (27%)
Excellent	7 (7%)

**Table 3:** Validity (AUC, sensitivity, specificity) of the CNR, SD BG, BG SNR, and liver SNR for predicting good image quality.

	**AUC**	**p**	**95% CI**	**Cutoff**	**Sensitivity**	**Specificity**	**PPV**	**NPV**
CNR	0.898	< 0.001⁣^∗^	0.831–0.964	> 26	82.35	78.79	66.7	89.7
SD BG	0.843	< 0.001⁣^∗^	0.762–0.924	≤ 7.7	76.47	75.76	61.9	86.2
BG SNR	0.859	< 0.001⁣^∗^	0.778–0.940	> 11.63	82.35	69.70	58.3	88.5
Liver SNR	0.744	< 0.001⁣^∗^	0.644–0.844	> 14	76.47	62.12	51.0	83.7

Abbreviations: AUC, area under the curve; CI, confidence interval; NPV, negative predictive value; PPV, positive predictive value; *p* value, probability value.

⁣^∗^Statistically significant at *p* ≤ 0.05.

**Table 4:** Univariate and multivariate linear regression analyses for the parameters affecting total readers (*n* = 100).

	**Total readers**				
	**r**	**Univariate**		^**#**^ **Multivariate**	
		**B** ** (LL–UL 95% CI)**	**p**	**B** ** (LL–UL 95% CI)**	**p**
SD BG	−0.668	−0.567 (−0.694 to −0.440)	< 0.001⁣^∗^	0.330 (0.125–0.535)	0.002⁣^∗^
Liver SD	−0.115	−0.120 (−0.327 to 0.087)	0.253		
BG SNR	0.790	0.332 (0.280–0.384)	< 0.001⁣^∗^	0.096 (−0.056 to 0.248)	0.214
Liver SNR	0.394	0.144 (0.077–0.212)	< 0.001⁣^∗^	0.017 (−0.031 to 0.065)	0.478
CNR	0.853	0.180 (0.158–0.202)	< 0.001⁣^∗^	0.203 (0.122–0.283)	< 0.001⁣^∗^

*Note:r*: Pearson coefficient; *B*: unstandardized coefficients.

Abbreviations: CI, confidence interval; LL, lower limit; UL, upper limit.

⁣^∗^Statistically significant at *p* ≤ 0.05.

^#^All variables with *p* < 0.05 were included in the multivariate analysis.

**Table 5:** Correlation between effective dose and BG SNR, liver SNR, and CNR (*n* = 100).

	**Effective dose**	
	**r**_**s**_	**p**
BG SNR	0.139	0.168
Liver SNR	0.100	0.323
CNR	0.072	0.479

*Note:r*_s_: Spearman coefficient.

**Table 6:** Relation between image quality and effective dose.

**Effective dose**	**Image quality**				**H**	**p**
	**Poor (** **n** = 5**)**	**Moderate (** **n** = 61**)**	**Good (** **n** = 27**)**	**Excellent (** **n** = 7**)**		
Mean ± SD	5.7 ± 1.8	4.9 ± 1.8	4.9 ± 1.9	5.8 ± 1.6	2.411	0.492
Median (min–max)	6.1 (3.6–7.8)	4.4 (2.4–7.9)	4.3 (2.1–7.9)	6.3 (2.7–8)		

*Note:H*: *H* for the Kruskal–Wallis test; *p*: *p* value for comparison between the studied categories.

## Data Availability

The datasets used and/or analyzed during the current study are available from the corresponding author on reasonable request of the International Journal of Biomedical Imaging.

## Nomenclature

ALARA: as low as reasonably achievable
BG: background
CNR: contrast-to-noise ratio
CT: computed tomography
HCC: hepatocellular carcinoma
HD: high definition
HU: Hounsfield unit
MDCT: multidetector computed tomography
MRN: medical record number
PACS: picture archiving and communication system
ROC: receiver operating characteristic
ROI: region of interest
SD: standard deviation
SNR: signal-to-noise ratio
WL: window level
WW: window width
